# Risk of High-Grade Cervical Lesions in Atypical Squamous Cells of Undetermined Significance (ASC-US) Cytology: Comparison between HIV-Infected and HIV-Negative Women

**DOI:** 10.31557/APJCP.2021.22.2.547

**Published:** 2021-02

**Authors:** Santipap Srisomboon, Charuwan Tantipalakorn, Tanarat Muangmool, Jatupol Srisomboon

**Affiliations:** *Department of Obstetrics and Gynecology, Faculty of Medicine, Chiang Mai University, Chiang Mai 50200, Thailand. *

**Keywords:** ASC-US, cervical cytology, HIV, uterine cervix, cervical screening results

## Abstract

**Background and objective:**

Women with human immunodeficiency virus (HIV) infection have an increased risk of HPV infection, cervical neoplasia. This study was undertaken to compare the risk of having high-grade cervical lesions defined as cervical intraepithelial neoplasia grade 2 or worse (CIN2+) in HIV-infected versus HIV-uninfected women who had atypical squamous cells of undetermined significance (ASC-US) on cervical cytology.

**Methods:**

Fifty-seven HIV-positive women aged 25-65 years with ASC-US cytology undergoing colposcopic examination between January 2008 and December 2020 at Chiang Mai University Hospital were reviewed. By matching 1:5 ratio, 285 HIV-negative women with ASC-US cytology in the same period were recruited as controlled subjects for comparison. The patient characteristics, HIV status, CD4 cell count within 6 months of colposcopy, antiretroviral therapy, parity, contraception, smoking history, number of sexual partners, and histopathology on cervical biopsy were analyzed.

**Results:**

Mean age ± SD of the HIV-positive and HIV-negative groups was 44.28 ± 8.53 years and 44.28 ± 9.68 years, respectively. HIV-positive women were significantly less likely to use contraceptive methods (36.8 % versus 48.8 % in HIV-negative women; P = 0.002). HIV-infected women significantly had more sexual partners than HIV-uninfected women. Both groups had similar risk for CIN 2+ (5.3 % in HIV-positive women compared with 4.9 % in HIV-negative women; odds ratio [OR] = 1.08, 95% confidence interval [CI] = 0.30 –3.87). After adjustment for no contraception use and number of sexual partners, the risk of CIN2+ in HIV-infected women remained unchanged; adjusted OR= 1.15, 95% CI = 0.27-4.92, P= 0.846).

**Conclusion:**

The risk of underlying high-grade cervical lesions in women with ASC-US on cervical cytology was approximately 5 %, regardless of HIV status.

## Introduction

Cervical cancer is the fourth most common malignancy in women worldwide with an estimated age-standardized incidence rate of 13.1/100,000 women-year. In 2018, there are approximately 570,000 new cases and 311,000 deaths (Bray et al., 2018). In Thailand, cervical cancer is the second most common female cancer after breast cancer with the estimated incidence of 16.2/100,000 women-year. Each year, 8,622 new cases are diagnosed and 5,015 die of cervical cancer (Bray et al., 2018). Cervical cancer is now recognized as the most preventable cancer because of various available methods of prevention, i.e. primary prevention with human papillomavirus (HPV) vaccination and secondary prevention with several screening tests including cervical cytology and HPV testing. 

Human immunodeficiency virus (HIV) infection increases the risk of acquiring several sexually transmitted infections. Women with HIV infection have an increased risk of co-infection with HPV, persistent HPV infection with multiple strains of HPV, cervical precancers and cancer (Palefsky et al., 1999; Ellerbrock et al., 2000; Abraham et al., 2013; Massad et al., 2015; Robbins et al., 2015). Since HIV-infected women are at higher risk of developing high-grade cervical lesions, i.e. cervical intraepithelial neoplasia (CIN) grade 2 and grade 3 (CIN2 and CIN3) and adenocarcinoma in situ (AIS) than those without HIV infection, women with HIV warrant careful cervical cancer screening and meticulous diagnostic work-up if abnormal screening results occur, with proper treatment for pathologically confirmed high-grade cervical lesions. 

In general, it is recommended that women with HIV infection should begin cervical cancer screening with cytology alone within 1 year of onset of sexual activity, or if already sexually active, within the first year after HIV diagnosis but no later than 21 years of age. Screening should continue throughout a woman’s lifetime, i.e. not stopping at age 65 years. If the initial cytology screening result is normal, the next cytology screening should be in 12 months. If the results of 3 consecutive annual cervical cytology screenings are normal, follow-up cervical cytology screening should be every 3 years. Co-testing (cervical cytology and HPV screening) is not recommended for HIV-infected women younger than 30 years. Women infected with HIV who are 30 years and older can be screened with cytology alone or co-testing (Moscicki et al., 2019). Although HPV testing plays a significant role in cervical cancer screening in developed countries, however, cervical cytology is still the principal available screening method in developing countries.

Among the cytologic abnormalities classified by the Bethesda system, atypical squamous cells of undetermined significance (ASC-US) is the most common (Kingnate et al., 2016). In general, management of women with an abnormal cervical cytology depends primarily on the severity of cytologic abnormality, which reflects the risk of having cervical precancerous and invasive lesions. In the American Society of Colposcopy and Cervical Pathology (ASCCP) guidelines, colposcopy is recommended for all HIV-infected women with ASC-US irrespective of CD4 count, HIV viral load, or antiretroviral therapy (Wright et al., 2002). It is interesting whether HIV-infected women with this mild cytologic abnormality are at similar or increased risk for precancerous cervical lesions compared with those who were HIV-uninfected. Accordingly, this study was conducted to compare the risk of underlying high-grade cervical lesions in HIV-infected versus HIV-uninfected women who had ASC-US cytology in cervical cancer screening. 

## Materials and Methods

After approval from the Research Ethics Committee, the data of women with ASC-US cytology undergoing colposcopic examination between January 2008 and December 2020 at Chiang Mai University Hospital (CMUH), were reviewed. It was the regulation of the hospital to check HIV status in every woman before undergoing colposcopy because of the high incidence of HIV infection in Chiang Mai province. In CMUH, colposcopy was carried out in all women with abnormal cytology due to the patient‘s anxiety and high default rate. Moreover, the expense for colposcopy procedure is much lower than that in the western countries. Colposcopic examinations were performed by gynecologic oncology fellows under supervision of the attending staff. Cervical biopsies and endocervical sampling were obtained at the discretion of the examiners. 

The inclusion criteria were women aged 25-65 years with ASC-US cytology on conventional Pap smears and available histopathology results. The exclusion criteria were women who were pregnant, had prior abnormal Pap smear, and history of cervical neoplasia, concurrent malignancy of other organs. The database includes patient characteristics, HIV status, CD4 cell count within 6 months of colposcopy, antiretroviral therapy, parity, contraception, smoking history, number of lifetime sexual partners, colposcopy findings, and histopathology on colposcopy-guided biopsy or subsequent diagnostic cervical excision and hysterectomy.

The sample size of this cross-sectional study was calculated by assuming the risk of having high-grade cervical lesions or worse (defined as CIN2+) of 33.3 % in HIV-infected women who had ASC-US cytology (Suwankanta et al., 2008) and 13.9 % in HIV-negative women with ASC-US smears (Kantathavorn et al., 2008). With set at 0.05 and at 0.20, the required sample size for HIV-positive cases was 57 women. HIV-negative women with ASC-US cytology were recruited as controlled subjects matching 5:1 for the following variables (± tolerance): age (± 5 years), and examination time period (± 6 months). The required numbers for HIV-negative controls were 285.

Descriptive statistics were used for demographic data. The Chi-square test for categorical variables and Mann-Whitney U test for continuous variables were used whenever appropriate to compare between the HIV-positive cases and HIV-negative controls. For factors with a P-value of less than 0.05 in univariate analysis, a multivariate analysis using binary logistic regression model was used to find the independent predictor. An odds ratio, with 95% confidence interval (CI) that did not include unity, was considered statistically significant. All statistical tests were 2 sided and a P-value of less than 0.05 was considered statistically significant.

## Results

Mean age ± SD and median age of the HIV-positive versus HIV-negative women were 44.28 ± 8.53 years versus 44.28 ± 9.68 years and 46 years versus 44 years, respectively. Of the 57 HIV- infected women, 56 (98.2%) received antiretroviral therapy. Two patients had opportunistic infections including tuberculous lymphadenitis (1) and pulmonary tuberculosis coexisting with Pneumocystis carinii pneumonia (1). The median CD4 cell count was 403.5 cells/microliter with a range of 300.0 – 571.0 cells/microliter. Two patients had CD4 cell count less than 200 cells/microliter. Characteristics of the 57 HIV-positive and 285 HIV-negative women who had ASC-US cytology are shown in Table 1. HIV-positive women were significantly less likely to use contraceptive methods (36.8 % versus 48.8 % in HIV-negative women; P = 0.002). Among the women who had used contraception, condom contraception was more common in HIV-positive women. HIV-infected women significantly had more sexual partners than HIV-uninfected women. 

HIV-infected women were significantly more likely to have CIN 1 or worse (54.4%) compared with 28.8% in HIV-uninfected women, crude odds ratio (OR) = 2.95, 95% CI =1.65-5.28; P = 0.004. After adjustment for factors found to differ significantly between the 2 groups in the initial analysis, i.e. contraception and number of sexual partners, the risk of having CIN 1 or worse in HIV-infected women was still significantly higher (Table 2).

The risk of having CIN 2 or worse on colposcopy-guided biopsy in HIV–positive women (3 of 57 or 5.3 %) was not significantly different when compared with that of HIV-negative women (14 of 285 or 4.9 %). The crude OR was 1.08, 95% CI = 0.30-3.87. After adjustment for the 2 factors (contraception and number of sexual partners) that were significantly different between the 2 populations, the odds of CIN 2 or worse in HIV-infected women compared with that of HIV-negative women remained unchanged (adjusted OR = 1.15, 95% CI = 0.27-4.92, P= 0.846) as shown in Table 2.

Invasive cervical cancer was detected in 2 patients, 1 in each group. One primiparous woman, aged 27 years, with HIV infection, had CIN3 on cervical biopsy. Loop electrosurgical excision procedure (LEEP) was performed and revealed invasive squamous cell carcinoma, 1 mm in depth and width with positive endocervical margin for CIN3. She was treated with simple hysterectomy showing residual CIN3 in endocervical canal of the specimen. The remaining 1 woman, aged 26 years, nulliparous without HIV infection had CIN3 on cervical biopsy. She underwent LEEP and had invasive squamous cell carcinoma, depth and width less than 1 mm with involved ectocervical margin for CIN1. Subsequent simple hysterectomy was carried out due to cancer phobia, severe pelvic pain from chronic pelvic inflammatory infection and no desire for future fertility preservation. No residual lesion was detected in the hysterectomy specimen. 

**Table 1:** Characteristics and Underlying Cervical Lesions of the Patients with ASC-US Cytology Categorized by HIV Status

Characteristics	HIV		P-value
	Positive (n=57)	Negative (n=285)	
Age (years)	46 (37-51)	44 (37-52)	0.886
Parity	1 (1-1)	1 (1-2)	0.061
Current smoker			0.864
No	56 (98.2)	279 (97.9)	
Yes	1 (1.8)	6 (2.1)	
Menopause			0.914
No	41 (71.9)	207 (72.6)	
Yes	16 (28.1)	78 (27.4)	
Contraception			0.002
No	36 (63.2)	146 (51.2)	
Hormone	5 (8.8)	61 (21.4)	
Tubal resection	6 (10.5)	59 (20.7)	
Condom	10 (17.5)	19 (6.7)	
Number of sexual partners	2 (2-4)	1 (1-2)	<0.001
Underlying lesions			<0.001
Normal	26 (45.6)	203 (71.2)	
CIN 1	28 (49.1)	68 (23.9)	
CIN 2+	3 (5.3)	14 (4.9)	

ASC-US, atypical squamous cells of undetermined significance; HIV, human immunodeficiency virus; CIN, cervical intraepithelial neoplasia; Data are present in median (range) and N (%); P values are performed using Chi-squared test for categorical variables and Mann-Whitney U test for continuous variables.

**Table 2:** Underlying CIN1 or Worse (CIN1+) and CIN2 or Worse (CIN2+) in Women with ASC-US cytology by HIV Status

Underlying	HIV		Odds Ratio (95%CI)		P
Lesions	Positive (%)	Negative (%)	Crude	Adjusted	
Normal	26 (45.6)	203 (71.2)	Reference	Reference	
CIN1+	31 (54.4)	82 (28.8)	2.95 (1.65-5.28)	2.61 (1.35-5.02)	0.004
Normal + CIN1	54 (94.7)	27 (95.1)	Reference	Reference	
CIN2+	3 (5.3)	14 (4.9)	1.08 (0.30-3.87)	1.15 (0.27-4.92)	0.846

CI, confidence interval; CIN, cervical intraepithelial neoplasia

## Discussion

In the present study, the risk of underlying high-grade cervical lesions in HIV-infected women with ASC-US on cervical cytology was 5.3 %, comparable to that of 4.9 % in HIV-uninfected women. The finding of equal risk for underlying high-grade cervical lesions in women with mildly abnormal cervical cytology regardless of HIV status supports the previous study comparing the prevalence of CIN 2 or worse (CIN2+) in a cohort of 72 HIV-positive and 360 HIV-negative women with mildly abnormal cervical cytology, i.e. ASC-US or low-grade squamous intraepithelial lesions (LSIL) cytology. Both populations were found to be at similar risk for CIN 2+ (15.2% in HIV-positive women compared with 13.3% in HIV-negative women; odds ratio = 1.17, 95% CI = 0.58 –2.39). Consequently, HIV–positive women were as likely as HIV-negative women to have high-grade cervical lesions on biopsy (Boardman et al., 2008). The lower risk of underlying CIN2+ in women with ASC-US cytology regardless of HIV status in our study compared to the study by Boardman et al., (2008) may result from different baseline characteristics of the studied population, i.e. number of lifetime sexual partners, smoking history, race/ethnicity, and antiretroviral therapy use. In addition, our study included only women with ASC-US on cervical cytology. Anderson et al., (2006) also noted that underlying cervical lesions did not differ by HIV infection in women with ASC-US cytology. The similar rate of CIN 2+ in both groups of women supports cytological screening in the HIV-infected population and suggests that HIV status alone does not confer an increased risk of harboring significant cervical diseases.

In contrast, several studies reported that the underlying cervical lesions may be more frequently detected in HIV-positive women who had mildly abnormal cytology compared with those without HIV infection. In a study of HIV-positive and HIV-negative women with mildly abnormal cervical cytology, HIV-infected women were approximately 4 times more likely to have CIN on biopsy, leading to the recommendation that all HIV-positive women with mildly atypical cells on cytology undergo colposcopic examination (Wright et al., 1996). In addition, another one study of HIV-negative and HIV-infected women evaluated for abnormal cervical cytology with colposcopy biopsy showing that 49% of the HIV-infected women had cervical lesions more severe than their cytology indicated, compared with 27% of the HIV-negative women (Fruchter et al., 1994). 

Our finding of similar risk for underlying CIN2+ in women with ASC-US cytology regardless of HIV status was different from the previous study conducted in the same institute which found that HIV-infected women with abnormal Pap smears were at higher risk of having significant cervical lesions, irrespective of severity of abnormal cervical smears (Suwankanta et al., 2008). The possible reasons for this difference was the immune status of HIV-infected women. In the previous study, only half of HIV-infected women received antiretroviral therapy (ART), while 98% of HIV-positive women in this study received ART. In addition, the median level of CD4 cell count in this study was notably higher than that in the previous study (403.5 vs 250 cells/microliter).

The prevalence of underlying CIN 2 or worse in women with ASC-US cytology in the present study was low at approximately 5 %, irrespective of HIV status. Our findings were different from an uncontrolled study in which high-grade cervical lesions were detected in 12% of 42 HIV-positive women with ASC-US smears (Kirby et al., 2004). This may result from different study population. Interestingly, 2 women with ASC-US smear in our study had underlying invasive cervical cancer, 1 case in each group. Other investigators in Thailand had previously reported that the risk of underlying cervical precancers in women with ASC-US cytology on cervical screening was quite high ranging from 8.0 % to 18.5 % (Kiatpongsan et al., 2006; Kantathavorn et al., 2008; Suntornlimsiri, 2010; Kingnate et al., 2016). Surprisingly, Thai women with ASC-US cytology were also at high risk of having invasive cervical cancer ranging from 1.7 % to 7.9 % (Kiatpongsan et al., 2006; Kantathavorn et al., 2008; Suntornlimsiri, 2010; Kingnate et al., 2016). In comparison, the risk of cervical cancer in women with ASC-US cytology in North American population was very low at approximately 0.1-0.2% (Wright et al., 2007). Accordingly, immediate colposcopy is recommended for Thai women with ASC-US cytology irrespective of HIV status, especially in women who are anxious about cancer and are expected to default follow-up. In addition, the expense of colposcopy in Thailand is much lower than that in the western countries.

Interestingly, the present study found that HIV-infected women with ASC-US cytology were significantly more likely to have CIN 1 or worse compared with HIV-uninfected women (54.4% versus 28.8%, P = 0.004). Women with HIV infection are more likely to have persistent HPV infection which is the main step of cervical carcinogenesis. A systematic review and meta-analysis on the impact of HIV on HPV natural history noted that HIV-positive women had higher HPV acquisition and lower HPV clearance than HIV-negative women (Liu et al., 2018). HPV infection was higher with declining CD4 cell count and was lower in those virally suppressed on ART. HIV-infected women had higher incidence of low-grade squamous intraepithelial lesions (LSIL) and high-grade squamous intraepithelial lesions (HSIL) largely because of the increase of HPV persistence. Some investigators noted that HIV-infected women were at increased risk of 4-5 times in developing CIN when compared with HIV-negative women who had high-risk sexual behaviors (Ellerbrock et al., 2000). Accordingly, cervical cancer screening is of particular importance for HIV-infected women.

In Thailand, cervical cytology is the main available method for cervical cancer screening.

Cervical cytology appears to be a reliable primary method for screening cervical cancer in HIV-infected women, although the data regarding the performance of this method are conflicting (Robinson et al., 2003; Ahdieh-Grant et al., 2004; Massad et al., 2012). Either conventional Pap smear or liquid-based cytology may be used for screening. There was no statistically significant difference in the detection rate of squamous intraepithelial lesions between conventional smears and liquid-based cytology in HIV-positive women (Swierczynski et al., 2004; Harris et al., 2005). Annual cytological screening appears to be reliable and safe for women infected with HIV. In a prospective study of 942 HIV-positive women who had 3 consecutive negative cytology results, after 15 months of follow-up, no woman developed precancerous cervical lesions. After 39 months of follow-up, precancerous cervical lesions were detected in only 2 % of women (Massad et al., 2012).

Limitation of the present study was the retrospective by nature, some data were not available, such as viral load, age at first sexual relation and HPV testing results that were not available in all HIV-infected cohort. Certain characteristics of both the HIV- infected and HIV- uninfected populations may also limit generalizability of the study. Furthermore, no data on long-term follow-up were available. The strength of our study was that all patients were treated at a single institution with same management protocol and all the cytopathologic specimens were examined by expert gynecologic pathologists.

In conclusion, the risk of having high-grade cervical lesions in HIV-infected women with ASC-US on cervical cytology was approximately 5 %, comparable to that in HIV-uninfected women. 

## References

[B1] Abraham AG, D’Souza G, Jing Y (2013). Invasive cervical cancer risk among HIV-infected women: a North American multicohort collaboration prospective study. J Acquir Immune Defic Syndr.

[B2] Ahdieh-Grant L, Li R, Levine AM (2004). Highly active antiretroviral therapy and cervical squamous intraepithelial lesions in human immunodeficiency virus-positive women. J Natl Cancer Inst.

[B3] Anderson JR, Paramsothy P, Heilig C (2006). Accuracy of Papanicolaou test among HIV-infected women. Clin Infect Dis.

[B4] Boardman LA, Cotter K, Raker C (2008). Cervical intraepithelial neoplasia grade 2 or worse in human immunodeficiency virus-infected women with mildly abnormal cervical cytology. Obstet Gynecol.

[B5] Bray F, Ferlay J, Soerjomataram I (2018). Global cancer statistics 2018: GLOBOCAN estimates of incidence and mortality worldwide for 36 cancers in 185 countries. CA Cancer J Clin.

[B6] Ellerbrock TV, Chiasson MA, Bush TJ (2000). Incidence of cervical squamous intraepithelial lesions in HIV-infected women. JAMA.

[B7] Fruchter RG, Maiman M, Sillman FH (1994). Characteristics of cervical intraepithelial neoplasia in women infected with the human immunodeficiency virus. Am J Obstet Gynecol.

[B8] Harris TG, Burk RD, Palefsky JM (2005). Incidence of cervical squamous intraepithelial lesions associated with HIV serostatus, CD4 cell counts, and human papillomavirus test results. JAMA.

[B9] Kantathavorn N, Kietpeerakool C, Suprasert P (2008). Clinical relevance of atypical squamous cells of undetermined significance by the 2001 bethesda system: experience from a cervical cancer high incidence region. Asian Pac J Cancer Prev.

[B10] Kiatpongsan S, Niruthisard S, Mutirangura A (2006). Role of human papillomavirus DNA testing in management of women with atypical squamous cells of undetermined significance. Int J Gynecol Cancer.

[B11] Kingnate C, Tangjitgamol S, Khunnarong J (2016). Abnormal uterine cervical cytology in a large tertiary hospital in Bangkok metropolis: Prevalence, management, and outcomes. Indian J Cancer.

[B12] Kirby TO, Allen ME, Alvarez RD (2004). High-risk human papillomavirus and cervical intraepithelial neoplasia at time of atypical squamous cells of undetermined significance cytologic results in a population with human immunodeficiency virus. J Low Genit Tract Dis.

[B13] Liu G, Sharma M, Tan N (2018). HIV-positive women have higher risk of human papilloma virus infection, precancerous lesions, and cervical cancer. AIDS.

[B14] Massad LS, D’Souza G, Tian F (2012). Negative predictive value of pap testing: implications for screening intervals for women with human immunodeficiency virus. Obstet Gynecol.

[B15] Massad LS, Xie X, D’Souza G (2015). Incidence of cervical precancers among HIV-seropositive women. Am J Obstet Gynecol.

[B16] Moscicki AB, Flowers L, Huchko MJ (2019). Guidelines for cervical cancer screening in immunosuppressed women without HIV infection. J Low Genit Tract Dis.

[B17] Palefsky JM, Minkoff H, Kalish LA (1999). Cervicovaginal human papillomavirus infection in human immunodeficiency virus-1 (HIV)-positive and high-risk HIV-negative women. J Natl Cancer Inst.

[B18] Robbins HA, Pfeiffer RM, Shiels MS (2015). Excess cancers among HIV-infected people in the United States. J Natl Cancer Inst.

[B19] Robinson WR, Luck MB, Kendall MA (2003). The predictive value of cytologic testing in women with the human immunodeficiency virus who have low-grade squamous cervical lesions: a substudy of a randomized, phase III chemoprevention trial. Am J Obstet Gynecol.

[B20] Suntornlimsiri W (2010). Women in a region with high incidence of cervical cancer warrant immediate colposcopy for atypical squamous cells of undetermined significance on cervical cytology. J Med Assoc Thai.

[B21] Suwankanta N, Kietpeerakool C, Srisomboon J (2008). Underlying histopathology of HIV-infected women with squamous cell abnormalities on cervical cytology. Asian Pac J Cancer Prev.

[B22] Swierczynski SL, Lewis-Chambers S, Anderson JR (2004). Impact of liquid-based gynecologic cytology on an HIV-positive population. Acta Cytol.

[B23] Wright TC, Jr., Cox JT, Massad LS (2002). 2001 consensus guidelines for the management of women with cervical cytological abnormalities. J Low Genit Tract Dis.

[B24] Wright TC Jr., Massad LS, Dunton CJ (2007). 2006 consensus guidelines for the management of women with abnormal cervical cancer screening tests. Am J Obstet Gynecol.

[B25] Wright TC Jr., Moscarelli RD, Dole P (1996). Significance of mild cytologic atypia in women infected with human immunodeficiency virus. Obstet Gynecol.

